# Challenges of Translational Science

**DOI:** 10.5935/abc.20170061

**Published:** 2017-05

**Authors:** Leonardo R. Garcia, Bertha F. Polegato, Leonardo A. M. Zornoff

**Affiliations:** Departamento de Clínica Médica - Faculdade de Medicina de Botucatu, Botucatu, SP - Brazil

**Keywords:** Animal Models, Ischemia-reperfusion injury, Myocardial Infarction, Signaling Pathways

The introduction of standardized therapies for the treatment of major cardiovascular diseases, such as heart failure and myocardial infarction, significantly reduced mortality. However, cardiovascular diseases are still one of the main causes of morbidity and mortality worldwide. Consequently, a large number of experimental studies are published regarding this subject. These studies investigate, in addition to the mechanisms involved in the genesis of cardiovascular disease, potential therapeutic targets, as well as interventions that are beneficial in reducing the size of the ischemic lesion and the progression of cardiac dysfunction and, consequently, decrease mortality.

In some situations, the results of preclinical research are reproducible in clinical studies. As an example, we could cite the influence of obesity on the process of cardiac remodeling. It is accepted that the remodeling process plays a critical role in the onset and progression of cardiac dysfunction secondary to different stimuli.^1^ Experimental studies have shown that obesity induces ventricular remodeling,^2^ since it has been confirmed in clinical studies.^3,4^

However, not infrequently, the success of the experimental treatments studied does not replicate when applied to clinical studies. In this sense, the analysis of some recently published papers in the *Arquivos Brasileiros de Cardiologia*, in the field of basic / experimental research exemplifies this phenomenon.

One of the most interesting topics in cardiology today are the strategies to attenuate ischemia / reperfusion injury (RI). Thus, in the rat model, hypothyroidism, associated with decreased levels of nitric oxide, protected the heart from IR injury. Similarly, physical exercise,^6^ administration of tramadol^7^ and consumption of nitrate^8^ were effective in decreasing IR-induced injury in the rat model. These and other positive results from experimental studies are obfuscated by the fact that to date, cardioprotection strategies in clinical studies have shown negative results.^9^

The reasons for this frustrating inconsistency between experimental and clinical studies are many and reflect the full complexity of translational research. The first difficulty that can be pointed out is in relation to the animals used in the experimental studies. We can observe that much of the research uses small animals, usually rodents, as the target of the intervention. It is well known that the physiology of the cardiovascular system of small rodents is not necessarily the same as that of humans. High heart rate and differences in cellular ion fluxes, including calcium flux, do not allow the extrapolation of the results of these studies to humans. In addition, small rodents used in laboratories are genetically very homogeneous and, in some situations, are virtually the same.^10^ Although the experimental model with large animals is more similar to human and large animals are genetically more heterogeneous, research involving models with larger animals is much more difficult to conduct.

Another point to highlight is that most of the experimental studies use young and healthy animals, which differs significantly from the reality of the patients included in the clinical studies. It is not uncommon for patients with cardiovascular disease to have more than one comorbidity. Even when comorbidities are inserted in the experimental models, they are not treated, as is the case with patients.^11^ Treatment of these comorbidities involves the use of several medications, such as angiotensin converting enzyme inhibitors and beta-blockers, which also exert a cardioprotective effect. Accordingly, some pathological modulating pathways of pathological processes may already be blocked, even partially, by such medications. Thus, the insertion of one more cardioprotective factor in clinical studies may lead to very subtle improvements in outcomes, which are not statistically significant. In addition, cardioprotection involves the activation of multifactorial mechanisms and the presence of comorbidities and medications can modify the individual panel of gene expression of patients.^12^

We must also consider that the most common experimental model to study the pathophysiological consequences of myocardial infarction is the external ligation of the anterior descending artery, whereas in humans, coronary occlusion is the result of a long inflammatory process. In this way, the activated signaling pathways can be completely different. Even when models of ApoE knockout mice are used in the induction of atherosclerosis, this happens in an artificial way and does not reproduce the reality of what happens in humans.^13^ Additionally, lipid metabolism in mice is different, since in mice there is a predominance of lipoprotein HDL, whereas in humans there is a predominance of LDL and VLDL.^13^

In addition, regarding the difficulties of reproducing experimental results in clinical studies in myocardial infarction, in humans one of the pillars of the treatment is the institution of reperfusion as soon as possible. This measure alone is already successful in decreasing infarct size and mortality, and the beneficial effect of any additional intervention can be minimized in clinical studies.^14^

Another limitation of translational research is the transposition of the doses used in animals to humans, as well as the time of onset and duration of treatment. The drug or substance should achieve adequate concentration in the target tissue, while at the same time it can not be excessive, because of the risks of side effects. As a consequence, subtherapeutic doses may sometimes be used in clinical studies. An example of this is the PREMIER study, which evaluated the effect of selective inhibitor of matrix metalloproteinases PG 116800 in patients after myocardial infarction. In this study, due to the risk of the onset of musculoskeletal syndrome, one of the side effects of the administration of inhibitors of matrix metalloproteinases, a dose lower than that shown to be effective in preclinical studies in pigs was used. Thus, despite promising therapy, this study did not show any effect of PG 116800 on clinical outcomes.^15^

Therefore, although the contributions of experimental research in the area of cardiology are unquestionable, the challenge that remains is getting a greater transposition, in the minimum of possible time, of the results obtained on the workbench to the clinical practice.
